# The mitigative effect of isorhamnetin against type 2 diabetes *via* gut microbiota regulation in mice

**DOI:** 10.3389/fnut.2022.1070908

**Published:** 2022-12-22

**Authors:** Jinjun Li, Huimin Yuan, Zhiqi Zhao, Li Li, Xiaoqiong Li, Liying Zhu, Xin Wang, Ping Sun, Yinping Xiao

**Affiliations:** ^1^State Key Laboratory for Managing Biotic and Chemical Threats to the Quality and Safety of Agro-Products, Zhejiang Academy of Agricultural Sciences, Hangzhou, China; ^2^Institute of Food Science, Zhejiang Academy of Agricultural Sciences, Hangzhou, China; ^3^Key Laboratory of Postharvest Preservation and Processing of Vegetables (Co-construction by Ministry and Province), Ministry of Agriculture and Rural Affairs, Hangzhou, China; ^4^School of Public Health, Shanxi Medical University, Taiyuan, China; ^5^Department of Pharmacology, School of Basic Medical Sciences, Shanxi Medical University, Taiyuan, China; ^6^Clinical Medicine College, Hangzhou Normal University, Hangzhou, China

**Keywords:** isorhamnetin, type 2 diabetes, fasting blood glucose, gut microbiota, lipid metabolism

## Abstract

In order to demonstrate the effects of isorhamnetin (IH) on the symptoms of type 2 diabetes mellitus (T2DM) and the role of gut microbiota in this process, an T2DM mouse model was established *via* a high-fat diet and streptozotocin. After 6 weeks of IH intervention and diabetes phenotype monitoring, the mice were dissected. We detected blood indicators and visceral pathology. Contents of the cecum were collected for 16S rRNA sequencing and short chain fatty acid (SCFAs) detection. The results showed that after IH intervention, the body weight of type 2 diabetic mice was gradually stabilized, fasting blood glucose was significantly decreased, and food intake was reduced (*P* < 0.05). Isorhamnetin significantly increased the level of SCFAs and decreased the levels of blood lipids and inflammatory factors in mice (*P* < 0.05). 16S rRNA sequencing results showed that *Lactobacillus* were significantly decreased and *Bacteroidales S24-7 group_norank* were significantly increased (*P* < 0.05). Interestingly, gut microbiota was significantly correlated with inflammatory factors, blood lipids, and SCFAs (*P* < 0.05). Taken together, our data demonstrated that isorhamnetin could improve the diabetic effects in T2DM mice, which might be mediated by gut microbiota.

## 1 Introduction

Type 2 diabetes mellitus (T2DM) is among the most common metabolic disorders and is mainly caused by defective insulin secretion by pancreatic β-cells and a loss of insulin sensitivity in target tissues (1). Nearly half a billion people are living with diabetes across the globe, and this number is predicted to reach 454 million (8.0%) by 2030 and 548 million (8.6%) by 2045 (2). Patients with T2DM are at an increased risk of developing various complications, such as cardiovascular disease, diabetic retinopathy, renal insufficiency, and leg ulcers, which have a major negative impact on their quality of life (3). Studies have shown that metabolic diseases, such as obesity and T2DM, are associated with chronic low-grade inflammation within adipose tissue, which eventually leads to adipose tissue dysfunction and an imbalance in systemic energy metabolism (4). Moreover, the gut microbiota are intricately implicated in the chronic low-grade inflammatory response, and barrier dysfunction is correlated with obesity, diabetes, and metabolic syndrome, exacerbating inflammation, which in turn leads to further barrier damage (5). Bidirectional regulation occurs between the gut microbiota and metabolic diseases, with microbiota-derived lipopolysaccharide causing low-grade inflammation, abnormalities in glycolipid metabolism, and subsequent development of metabolic syndrome (6). Of the 380 million patients with diabetes worldwide, about 120 million use metformin, which is among the most popular oral hypoglycemic agents and first-line treatment against T2DM (7, 8). However, it is inevitably linked to adverse side effects (9). Hence, there is an incentive to develop natural compounds for the prevention and treatment of diabetes without side effects.

As important secondary metabolites in plants, flavonoid compounds have a positive impact on human and animal health, preventing chronic disease and having significant anti-inflammatory as well as antioxidant activity (10). They can inhibit the expression of pro-inflammatory factors and hence, are considered natural inhibitors of inflammation (11). Isorhamnetin (IH) is a flavonoid predominantly found in sea buckthorn and ginkgo fruits, with a variety of pharmacological effects, including anti-inflammatory, anti-tumor, antioxidant, antibacterial, and antiviral effects (12). In addition, IH exerts protective effects against cardiovascular diseases (13) and a variety of tumors (14), and has potential preventive effects in neurodegenerative diseases, such as Alzheimer’s disease (15). Cactus extracts rich in IH glycosides were suggested to prevent the development of dietary-induced obesity-related metabolic abnormalities (16).

Several studies have used rodent models to study the anti-diabetic effects of IH. Matboli et al. found that treatment with IH at three different doses showed a significant decrease in m-TOR, IGF1-R, and LncRNA-RP11-773H22.4 and up-regulated the expression of *Akt2* mRNA, miR-1, and miR-3163 in adipose tissue (17). IH can also inhibit the signaling activity of NF-κB, reduce the production of inflammatory mediators, reduce oxidative stress in diabetic rats, and improve the kidney injury caused by diabetes (18). Another study found that IH promotes glucose uptake and may have the beneficial function of maintaining glucose homeostasis by preventing hyperglycemia at physiological concentrations. However, the effect of IH on gut microbiota remains elusive. Hence, we aimed to determine the impact of IH on fasting blood glucose levels, body weight, water intake, food intake, serum lipid profile, and inflammation in mouse models of T2DM, in addition to changes in short-chain fatty acids and gut microbiota structure. Based on the above results, we also analyzed the correlation between gut microbiota and host metabolism.

## 2 Materials and methods

### 2.1 Experimental animals and treatments

Thirty-six healthy, male C57BL/6J mice (3 weeks old, specific-pathogen-free) were purchased from Shanghai SLAC Laboratory Animal, Co., Ltd. [License No.: SCXK (Shanghai) 2017-0005]. Animal experiments were carried out in the Laboratory Animal Center of Zhejiang Academy of Agricultural Sciences [Animal Experimentation License No.: SYXK (Zhejiang) 2020-0022]. The study was approved by the Zhejiang Ethics Committee for Animal Experiments (ethics approval number: 2021ZAASLA50). All experimental animals were given 7 days to adapt to the animal room conditions and were then randomly divided into the control NC group (6 mice) and the T2DM group. The NC group consisted of 6 C57BL/6J mice fed a general maintenance diet throughout the entire study period. The T2DM group (30 mice) mice were fed a custom high-fat, high-sugar diet (composition: 60% fat, 20% carbohydrate, 20% protein, diet type: D12492, supplied by Jiangsu Xietong Medical Biological Engineering Co., Ltd. China). Both the NC and T2DM groups were given purified water. After 6 weeks, the T2DM group mice received an intraperitoneal injection of streptozotocin solution (80 mg/kg in 0.1 M sodium citrate buffer, pH 4.2–4.5) daily, for 2 days (19). The NC group mice received injections of 0.1 M sodium citrate buffer. After 5 days, the FBG levels were measured following a 12-h fast. The diabetes mouse model was considered successfully established when the fasting blood glucose level was ≥11.1 mmol/L. These T2DM mice were switched to a Co60-irradiated maintenance diet until the end of the experiment. The T2DM mice were randomly divided (6 mice per group) into the T2DM group, metformin treatment positive control group (100 mg/kg administered by gavage, PC), IH low dosage group (75 mg/kg administered intragastrically, IH75), IH medium dosage group (150 mg/kg, IH150), and IH high dosage group (300 mg/kg, IH300).

### 2.2 Measurement of blood glucose level, body weight, and water and food intakes

After 12 h of fasting, body weight and FBG levels were measured at a fixed time each week. FBG levels were determined by tail blood sampling as required by the glucose meter and glucose test strip (20). Food intake and water intake for the week were measured at 9 p.m. the day before the fast.

### 2.3 Lipid profiling

Triglyceride (TG) levels, cholesterol (CHO) content, high density lipoprotein cholesterol (HDL-C) content, and low-density lipoprotein cholesterol (LDL-C) content were determined using the GPO-PAP/endpoint method, CHOD-PAP/endpoint method, direct method-selective inhibition method-endpoint method, and the direct method-surfactant removal method-endpoint method, respectively (21).

### 2.4 Measurement of serum IL-1β levels

Serum IL-1β concentrations were quantitatively determined *via* enzyme-linked immunosorbent assay. Whole blood samples were stored at room temperature for 2 h, then centrifuged at 2400 rpm for 15 min at 4°C, and the supernatants were collected. The IL-1β concentrations were measured using the elisa kit (Wuhan Saipei Biotechnology Co., Ltd., China) according to the manufacturer’s instructions.

### 2.5 Measurement of liver and kidney weight indices

After the mice were dissected, the liver and kidney tissues were weighed. The ratio of liver and kidney weight to body weight was calculated to obtain the liver and kidney indices.

### 2.6 Histopathological hematoxylin and eosin (H&E) staining

Mice received intragastric administration of IH for 6 weeks and were then dissected after anesthesia with isoflurane. The collected blood samples were centrifuged at 2400 rpm for 10 min at 4°C, and serum was obtained for further testing. Collected livers and kidneys were immersed in 4% paraformaldehyde tissue fixative, embedded in paraffin, and were sectioned using an RM2016 pathological (Hangzhou Servicebio Technology Co., Ltd. China) microtome to obtain 4 μm-thick sections. The sections were deparaffinized, stained with hematoxylin and eosin, dehydrated, and mounted. Histopathological changes were observed and imaged at 200× magnification using a Nikon Eclipse E100 high-power microscope and Nikon DS-U3 imaging system.

### 2.7 Measurement of SCFAs

Mouse cecal contents samples were removed after dissection and stored at −80°C for future use. The cecal samples were diluted (1:10) with sterile phosphate buffered saline solution and vortexed. The mixture was then centrifuged at 6000 rpm for 6 min, and the supernatant was collected and transferred to a sterile centrifuge tube. Two hundred fifty microliters of supernatant was pipetted into another sterile container, and 50 μL crotonic acid solution was added to the supernatant at a ratio of 1:5. The mixture was then filtered through a 0.22 μm membrane, acidified, and stored at −80°C for later use. The samples were then tested using a gas chromatograph (GC-2010 Plus; Shimadzu Corporation, Kyoto, Japan) equipped with a DB-FFAP column (Agilent Technologies, Inc., Santa Clara, CA, USA).

### 2.8 Gut microbiota analysis

After laparotomy was performed on the mice to collect blood samples, the cecum contents was quickly cut out, immersed in liquid nitrogen for rapid freezing, and stored at −80°C for later use. 16SrRNA sequencing was performed by Mingke Biotechnology Co., Ltd. (Hangzhou, China). The amplified regions were sequenced using the Illumina HiSeq platform, and QIIME2 software was used to analyze the sequencing results. The OTUs were demarcated from the non-repeating sequences at a similarity threshold of 97% and subjected to further analysis. Representative sequences of all OTUs were matched against the Silva and RDP databases to identify species. Principal component analysis (PCA) was performed to analyze inter-sample beta diversity.

### 2.9 Statistical analysis

SPSS 23.0 and GraphPad Prism 8 were used for data processing and data analysis. The study data are presented as the mean ± SEM. Multiple-group comparisons were performed using one-way ANOVA and non-parametric tests. The Student-Newman-Keuls method was used for multiple comparisons between the means of each group. Correlation was determined *via* Pearson’s linear correlation analysis. A *P*-value < 0.05 was considered to statistically significance.

## 3 Results

### 3.1 Impact of IH on the body weight, fasting blood glucose (FBG) levels, and food and water intake of mice

**FIGURE 1:**
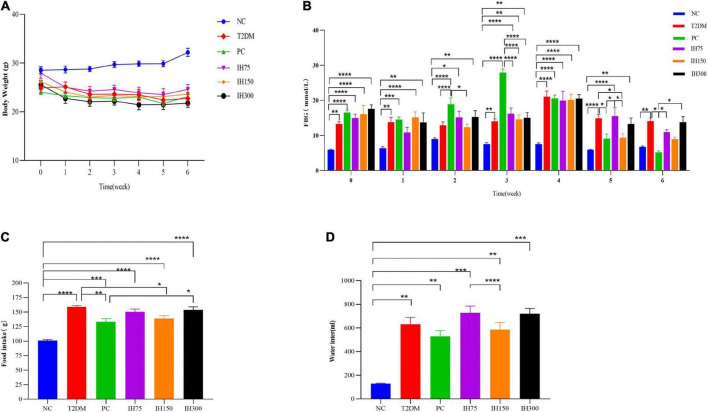
Effects of isorhamnetin (IH) on body weight **(A)**, fasting blood glucose **(B)**, food intake **(C)**, and water intake **(D)** in mice. All data are expressed as mean ± standard error of the mean (SEM). *N* = 6, *P* < 0.05 (*), *P* < 0.01 (**), *P* < 0.001 (***), and *P* < 0.0001 (****).

As shown in Figure 1A and Supplementary Figure 1A, the body weight of the T2DM group mice decreased gradually (*P* < 0.05) until the body weight stabilized starting from the third week (*P* < 0.01). In weeks 5–6, the body weight of the IH75 and IH150 groups showed an increasing trend (*P* > 0.05).

Details on the FBG levels are shown in Figure 1B. There were significant differences in FBG levels between the NC and the other groups at week 0 (*P* < 0.01). In the first week, all the other groups except IH75 had significant differences in FBG levels compared with the NC group (*P* < 0.01). In contrast, IH75 treatment delayed the early rise in blood sugar. At the second week, there was no significant differences in FBG levels between the NC, T2DM, and IH75 groups. The FBG levels of the IH150 group was significantly lower than that of the PC group (*P* < 0.05). In the third week, there were significant differences in FBG levels between the NC group and the other groups (*P* < 0.01), and significant differences between the PC group and the IH and NC groups (*P* < 0.0001). Relatively speaking, the IH150 treatment had better hypoglycemic effects. At the fourth week, there were significant differences in the FBG levels between the NC group and other groups (*P* < 0.0001). At week 5, the FBG levels of the NC group had no significant difference from that of the PC and IH150 groups, but had significant difference from that of the other groups (*P* < 0.01). Compared with the T2DM group, the FBG levels in the PC and IH150 groups was significantly decreased (*P* < 0.05). At week 6, the FBG levels of the T2DM group was significantly higher than that of the NC group (*P* < 0.01). The NC group had no significant difference with the PC, IH75, IH150, and IH300 groups (*P* > 0.05) in their FBG levels. The FBG levels in the PC group were significantly decreased compared to that of the T2DM group (*P* < 0.05). The FBG levels of the IH75 and IH300 groups were significantly increased compared with that of the PC group (*P* < 0.01), but the IH150 group had no significant difference compared with that of the PC group (*P* > 0.05). In general, the PC and IH150 treatments had stronger hypoglycemic effects (*P* < 0.05).

As shown in Figure 1C, the food intake of the other groups was significantly higher than that of the NC group (*P* < 0.001). The food intake of the PC and IH150 groups were significantly lower than that in T2DM group (*P* < 0.05).

The water intake (Figure 1D) of the other groups was significantly higher than that of the NC group (*P* < 0.01). The water intake of the IH75 group was significantly higher than that of the IH150 group (*P* < 0.0001). In general, the PC and IH75 treatments have the potential to improve the polydipsia of T2DM mice.

### 3.2 Impact of IH on serum indices in mice and correlation analysis

**FIGURE 2:**
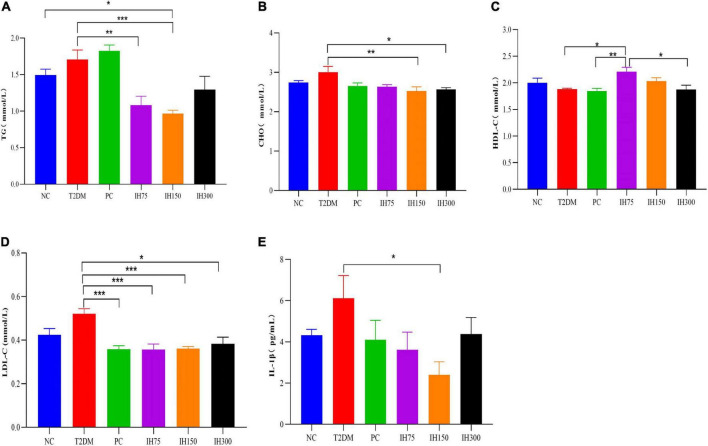
Effect of IH on serum triglycerides (TG) **(A)**, cholesterol (CHO) **(B)**, high density lipoprotein cholesterol (HDL-C) **(C)**, low density lipoprotein cholesterol (LDL-C) **(D)**, and interleukin (IL)-1β **(E)** in mice. All data are expressed as mean ± standard error of the mean (SEM). *N* = 6, *P* < 0.05 (*), *P* < 0.01 (**), and *P* < 0.001 (***).

As shown in Figure 2A, the TG levels of the IH75 and IH150 groups decreased significantly compared with that of the T2DM group (*P* < 0.01). There are also significant differences between the IH150 and NC groups. Serum CHO levels in the IH150 and IH300 groups (Figure 2B) were significantly lower than those in T2DM mice (*P* < 0.05). As shown in Figure 2C, the HDL-C levels in the IH75 group were significantly higher than that in the T2DM, PC, and IH300 groups (*P* < 0.05). The PC, IH75, IH150, and IH300 mice had significantly lower serum LDL-C levels than those of the T2DM group (*P* < 0.01) (Figure 2D). In addition, the IH150 group mice had significantly lower serum IL-1β levels than their T2DM group counterparts (*P* < 0.05) (Figure 2E).

### 3.3 Impact of IH on liver and kidney weight indices and pathological changes in experimental mice

#### 3.3.1 Liver and kidney weight indices

**FIGURE 3:**
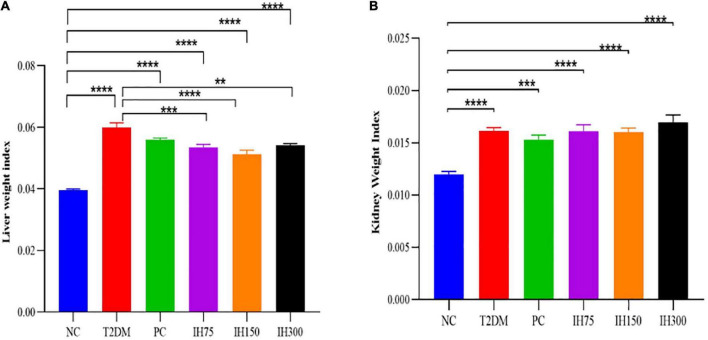
Effects of IH on liver weight index **(A)** and kidney weight index **(B)** in mice. All data are expressed as mean ± standard error of the mean (SEM). *N* = 6, *P* < 0.01 (**), *P* < 0.001 (***), and *P* < 0.0001 (****).

The liver weight indices (Figure 3A) of the other groups were significantly higher than that of the NC group (*P* < 0.0001). Among them, the indices of the IH75, IH150, and IH300 groups were significantly lower than that of T2DM mice (*P* < 0.01). The kidney weight index (Figure 3B) of all the other groups was significantly higher than that of the NC group (*P* < 0.001).

#### 3.3.2 Pathological changes

**FIGURE 4:**
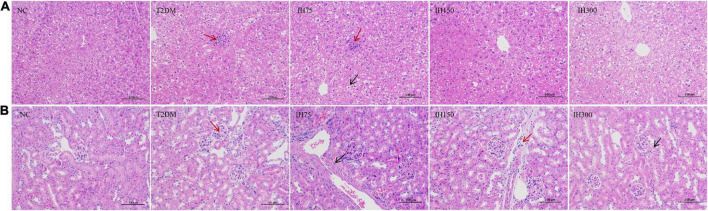
Effects of IH on pathological changes in mice. Scale bar = 100 μm. Representative H&E staining of liver **(A)** and kidney **(B)** tissue sections.

Granular degeneration in hepatocytes, in addition to a loose, lightly stained, and granular cytoplasm were observed around the central vein and portal area as well as in the liver parenchyma of mice from the NC group. No obvious inflammatory changes were observed. These alterations were also noted in mice of the T2DM group; however, these mice also exhibited occasional lymphocyte infiltration around blood vessels (red arrows, Figure 4A). Hepatocyte ballooning, centrally located nuclei, and a vacuolated cytoplasm were observed in the hepatic tissues of mice from the IH75 group (black arrows, Figure 4A), in addition to occasional focal inflammatory cell infiltration (red arrows, Figure 4A). The same alterations were noted in mice of the IH150 and IH300 groups, with the exception of the inflammatory cell infiltration. Representative images are shown in Figure 4A.

Evenly distributed glomeruli and cells, and stroma within the glomeruli, in addition to closely arranged tubules and no obvious stromal hyperplasia nor inflammatory alterations were observed in mice of the NC group. Vacuolar degeneration in a small number of renal tubule epithelial cells and small, round cytoplasmic vacuoles were observed in mice of the T2DM group, with focal lymphocytic infiltrations around local blood vessels (red arrows, Figure 4B). The same structures were noted in IH75, IH300, and IH150 mice (black arrows, Figure 4B), with the exception of the inflammatory cell infiltration in the former two groups and some scattered inflammatory cells around vessels in the latter group. Representative images are shown in Figure 4B.

### 3.4 Impact of IH on the gut microbiota of experimental mice

#### 3.4.1 Analysis of gut microbiota structure

**FIGURE 5:**
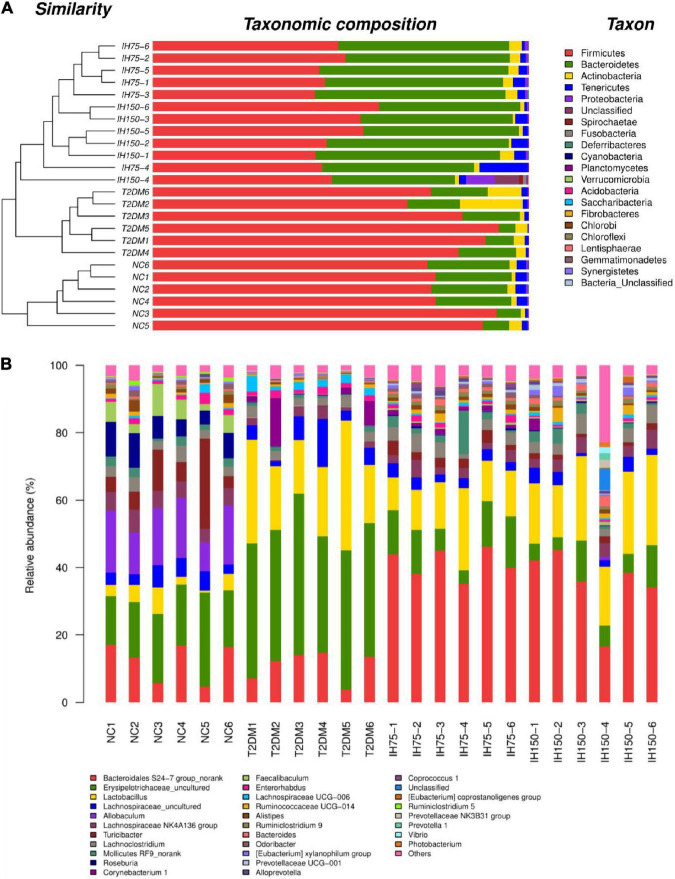
Effect of IH on bacterial microbiota composition **(A)** dendrogram with bar graph of phyla; **(B)** relative abundance of genera.

As can be seen from the analysis of microbial community structure and the dendrogram, the dominant phyla in each group mainly consisted of Firmicutes, Bacteroidetes, Actinobacteria, Tenericutes, and Proteobacteria. However, mice in the T2DM group had higher proportions of Firmicutes and Bacteroidetes than the NC group mice. Low or medium doses of IH decreased the proportions of these two phyla (Figure 5A). Dominant genera mainly included *Bacteroidales S24-7 group_norank, Erysipelotrichaceae_uncultured, Lactobacillus, Lachnospiraceae_uncultured, Allobaculum, Lachnospiraceae NK4A136 group, Turicibacter*, and *Lachnoclostridium*. Low or medium doses of IH increased the proportion of *Bacteroidales S24-7 group_norank* and *Lactobacillus*, while lowering that of *Erysipelotrichaceae_uncultured*. Both *Erysipelotrichaceae_uncultured* and *Lactobacillus* increased in the T2DM group (Figure 5B). Overall, the above results suggested that IH can improve gut microbiota abundance and diversity in T2DM mice.

#### 3.4.2 Analysis of mouse gut microbiota alpha-diversity

**FIGURE 6:**
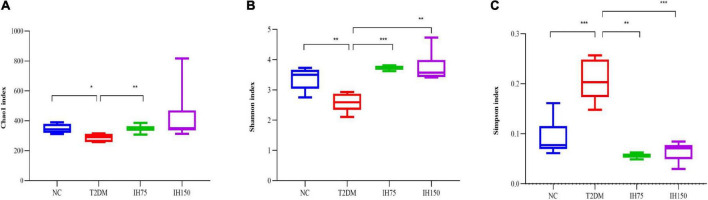
Effect of IH on alpha-diversity. Chao1 index **(A)**, Shannon index **(B)**, Simpson index **(C)**. All data are expressed as mean ± standard error of the mean (SEM). *N* = 6, *P* < 0.05 (*), *P* < 0.01 (**), and *P* < 0.001 (***).

Analysis of gut microbiome abundance indicated that the Chao index of the T2DM group was significantly lower than that of the NC (*P* < 0.05) and IH low dosage (*P* < 0.05) groups (Figure 6A). Gut microbiome diversity analysis revealed that the Shannon index of the T2DM group mice was significantly lower than that of mice from the NC, low dosage, and medium dosage groups (*P* < 0.01) (Figure 6B). The Simpson index of the T2DM group mice was significantly higher than that for all other groups (*P* < 0.01), indicative of lower gut microbiome diversity in these animals (Figure 6C).

#### 3.4.3 Analysis of beta-diversity

**FIGURE 7:**
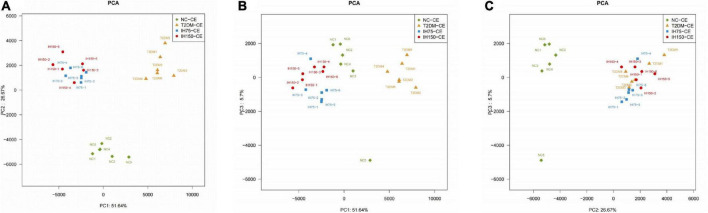
Principal coordinate analysis (PCA) of the bacterial structure of the mouse cecal contents. **(A–C)** Are PCA from different perspectives.

**FIGURE 8:**
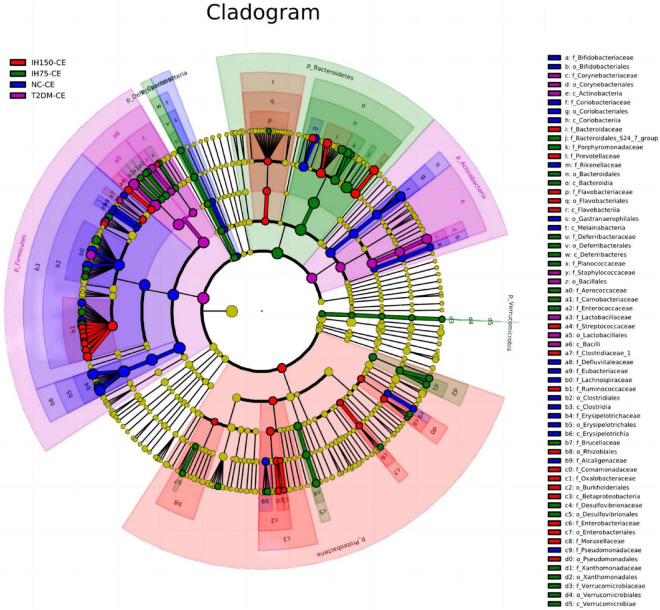
Clustering tree of cecal microbiota. The different color nodes represent different groups. The species names indicated by the English letters in the picture are shown in the legend on the right.

**FIGURE 9:**
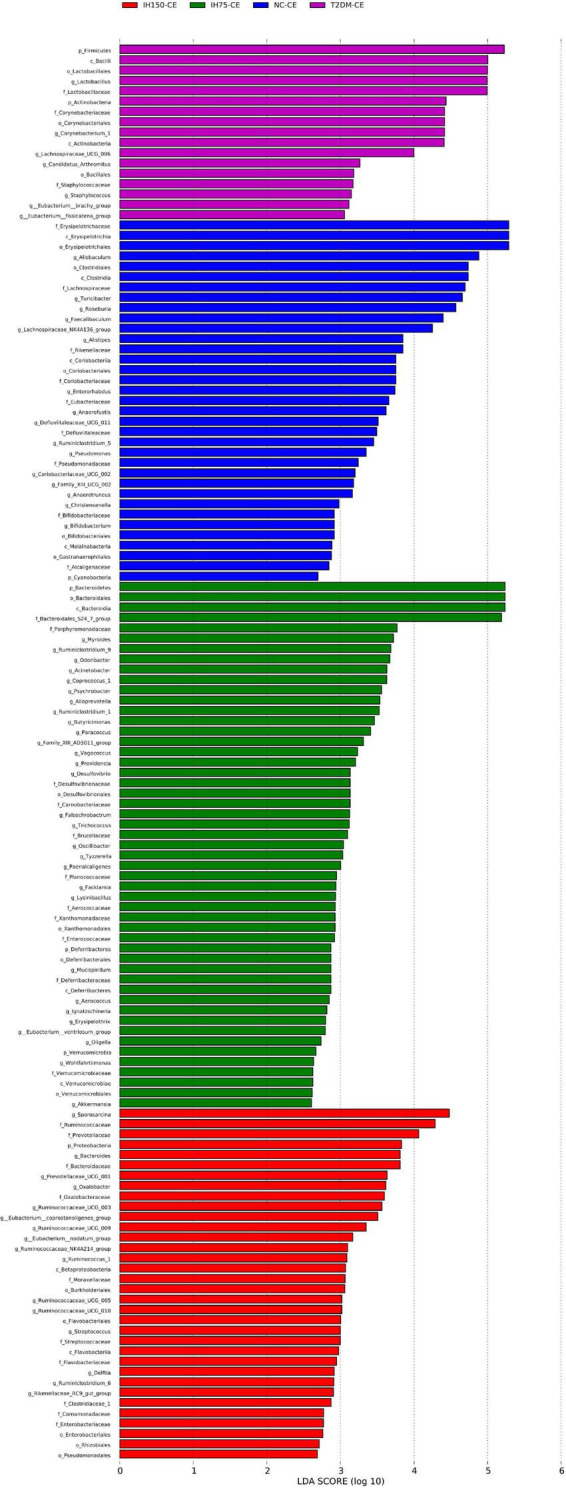
Linear discriminant analysis (LDA) score of the statistically significant microbial groups in the two groups.

Principal component analysis (PCA) suggested that the T2DM group had significantly different clustering from all other groups. The IH low and medium dosage groups also had distinguishable clusters, similar to those of the NC group. Non-metric multidimensional scaling based on the beta-diversity distance revealed relatively significant inter-group differences, but the IH intervention groups were close to the NC group (Figure 7). Linear discriminant analysis effect size was used to analyze the statistically significant differences in species and biological correlation (Figures 8, 9). The non-parametric factorial Kruskal–Wallis (KW) sum-rank test was first performed to determine taxa with significantly differential abundance. Finally, linear discriminant analysis (LDA) was employed to estimate the impact of the abundance per component (species) on differences. LDA > 2 and *P* < 0.05 were adopted as a threshold for the screening of differentially abundant species, and a total of 137 species were identified. In the NC group, Firmicutes.c_Erysipelotrichia, Firmicutes.c_Clostridia, Firmicutes.c_Erysipelotrichia, Actino- bacteria.c_Coriobacteriia, and Bacteroidetes.c_Bacteroidia were the dominant classes of bacteria. In the T2DM group, Firmicutes.c_Bacilli, Actinobacteria, and Firmicutes.c_Clos- tridia were dominant. In the IH75 group, Bacteroidetes.c_Bacteroidia, Firmicutes.c_Clostridia, and Proteobacteria.c_Deltaproteobacteria were the most dominant. In the IH150 group, Firmicutes.c_Clostridia, Bacteroidetes.c_Bacteroidia, and Proteobacteria were the dominant classes of bacteria.

### 3.5 Impact of IH on short-chain fatty acids (SCFAs) levels in mice

**FIGURE 10:**
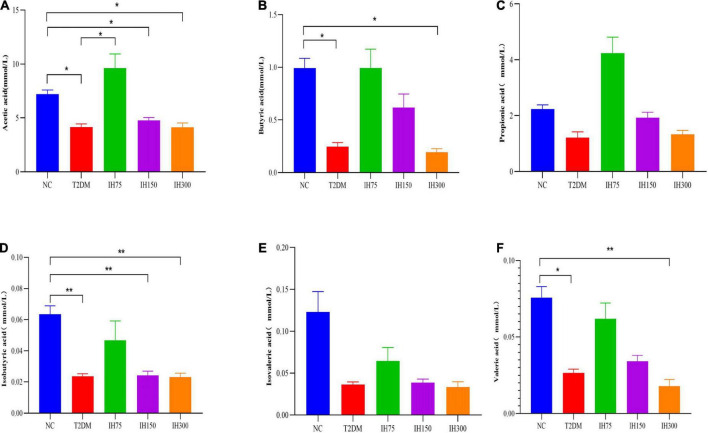
Effect of IH on short-chain fatty acids in mice. Acetic acid **(A)**, butyric acid **(B)**, propionic acid **(C)**, isobutyric acid **(D)**, isovaleric acid **(E)**, and valeric acid **(F)**. All data are expressed as mean ± standard error of the mean (SEM). *N* = 6, *P* < 0.05 (*) and *P* < 0.01 (**).

Acetic acid levels in the T2DM group were significantly lower than that in the NC group (*P* < 0.05). Further, medium and high doses of IH induced a significant reduction in acetic acid levels relative to those in the NC group (*P* < 0.05). However, mice in the IH low dosage group exhibited significantly elevated acetic acid concentrations in their cecal contents, with no significant difference relative to the NC group (*P* > 0.05), while being significantly higher than that in the T2DM group (*P* < 0.05) (Figure 10A). Butyric acid levels in the cecal contents of mice from the T2DM model group were lower than those in the NC group (*P* < 0.05). However, the IH low and medium dosage groups experienced elevated butyric acid levels after treatment, without significant differences relative to the NC group (*P* > 0.05). High-dose IH induced a significant reduction in butyric acid levels compared to the NC group (*P* < 0.05) (Figure 10B). No statistically significant inter-group differences in the cecal propionic acid concentrations were noted (*P* > 0.05). The T2DM group had lower propionic acid levels, whereas the IH low dosage group exhibited significantly elevated propionic acid levels following treatment. Among the different dosages tested, higher IH doses contributed to less significant increases in propionic acid levels (Figure 10C). The cecal isobutyric acid levels of mice from the T2DM model group were significantly lower than those of NC group mice (Figure 10D). However, low-dose IH increased these levels, with no significant difference from the NC group (*P* > 0.05). More specifically, isobutyric acid levels in the T2DM, IH150, and IH300 groups were significantly lower than that in the NC group (*P* < 0.01), without significant inter-group differences in the levels of isovaleric acid (*P* > 0.05) (Figure 10E). The cecal isovaleric acid concentration of mice in each T2DM model group was lower than that in the normal group, with low-dose IH relatively increasing isovaleric acid levels. The cecal valeric acid concentration of mice from each T2DM model group was also lower than that in the NC group. Further, the valeric acid level in the NC group was significantly higher than that in the T2DM group (*P* < 0.05). Low-dose IH increased the cecal valeric acid levels of T2DM model mice, while levels in the IH high dosage group were significantly lower than that in the NC group (*P* < 0.01). Valeric acid levels in the IH75, IH150, and IH300 groups exhibited a decreasing trend with gradient variations (Figure 10F).

### 3.6 Correlation between SCFAs and gut microbiota

Correlation analysis for the butyric acid level with the Shannon and Simpson indices revealed significant correlations (*P* < 0.05). Higher levels of butyric acid were associated with higher Shannon indices and lower Simpson indices. These results suggested that an increase in butyric acid is conducive to higher gut microbiome diversity. Acetic acid levels were not correlated with the Shannon index, but were correlated with the Simpson index (*P* < 0.05). Higher propionic acid levels were also correlated with higher Shannon and lower Simpson indices (*P* < 0.05). Overall, greater SCFA levels were conducive to intestinal health. Correlation analysis for *Lactobacillus* abundance with acetic, butyric, and valeric acid levels yielded significant results (*P* < 0.05). Furthermore, higher levels of *Lactobacillus* were associated with lower levels of all three SCFAs. Correlation analysis for the *Bacteroidales S24-7 group_norank* abundance with acetic and propionic acid levels yielded significant correlation (*P* < 0.05). Greater *Bacteroidales S24-7 group_norank* abundance was associated with higher acetic and propionic acid levels (Supplementary Figures 1, 2).

### 3.7 Correlation between gut microbiota and biochemical indices

Correlation analysis revealed that *Bacteroidales S24-7 group_norank* abundance had significant negative correlation with IL-1β and cholesterol levels (*P* < 0.05). That is, a higher abundance of *Bacteroidales S24-7 group_norank* was accompanied by lower IL-1β and cholesterol levels, which suggested that *Bacteroidales S24-7 group_norank* was conducive to the health of T2DM mice. The relative abundance of *Bacteroidales S24-7 group_norank* in the IH75 and IH150 groups was higher than that in the NC and T2DM groups (*P* < 0.05) (Supplementary Figures 1, 2).

## 4 Discussion

Type 2 diabetes mellitus is a metabolic disease characterized by insulin resistance or defective insulin secretion by pancreatic β-cells (16). Polydipsia, polyphagia, polyuria, and emaciation are considered typical symptoms of T2DM and their presence is among the diagnostic criteria (16). In mice, a fasting blood glucose ≥11.1 mmol/L is the diagnostic criterion for T2DM (22). T2DM can alter glycolipid metabolism in patients (23). Hence, we chose to assess water intake, food intake, fasting blood glucose, and body weight as relevant physiological indices, in addition to serum TG, CHO, HDL-C, and LDL-C levels as well as biochemical indices of the lipid profile. During the early stages of intragastric IH administration, the T2DM model mice developed severe polydipsia, polyphagia, and polyuria, exhibiting a sharp decrease in body weight relative to mice of the NC group. After intragastric administration of IH for 6 weeks, the T2DM symptoms of mice, including polyphagia and polydipsia, improved, contributing to an overall upward trend in body weight. This upward tendency was not observed in the high dosage group. As can be inferred from the FBG level data, IH ameliorated glycometabolism in T2DM mice. In contrast to mice of the T2DM group, the IH75 and IH150 group mice experienced a decrease in serum TG and LDL-C levels as well as an increase in HDL-C levels. The IH150 group mice exhibited a drop in serum cholesterol and IL-1β levels. Some studies have found that high-fat diet (HFD) and inflammation are key contributors to insulin resistance and T2DM. Interleukin (IL)-1β plays a role in insulin resistance and sensitivity through tumor necrosis factor-independent and dependent pathways (24). It is possible to reduce blood sugar by improving lipid distribution and regulating lipid metabolism (25). Inhibition of hepatic adipogenesis and gluconeogenesis can promote glycogen synthesis (26). IH can reduce the production of inflammatory mediators and improve the kidney damage associated with T2DM (18). IL-1β and insulin increased the uptake of glucose into macrophages, which identify a physiological role for IL-1β and insulin in the regulation of both metabolism and immunity (27). These results indicated that low or medium doses of IH might be conducive to lowering blood lipid indices and alleviation of inflammation in patients with T2DM. Pathological analysis of liver and kidney sections revealed that 6 weeks of IH intervention exerted protective effects in these organs in T2DM mice.

To date, the impact of gut microbiota on T2DM has increasingly attracted public attention, and changes in gut microbiota are believed to be associated with diabetes (28). Gut microbiota dysbiosis can lead to a decrease in beneficial bacterial groups and/or an increase in opportunistic pathogenic bacteria, thereby inducing chronic low-grade intestinal inflammation (29). Previous studies suggested that diabetic patients have a significantly reduced amount of *Bifidobacteria*, *Bacillus fusiformis* (30–32), and Bacteroidetes (33) among their gut microbiota when compared with healthy people. Gut dysbiosis can also lead to a greater number of operational taxonomic units (OTUs) and a higher percentage of *Lactobacillus* in the gut of obese, diabetic mice (34). Hence, improving the gut microbiota is conducive to the prevention and treatment of T2DM. Therefore, we evaluated the impact of IH on gut microbiota within the cecum of mice with T2DM induced by a high-fat, high-sugar diet and streptozotocin. It was previously suggested that T2DM is associated with chronic low-grade inflammation in adipose tissue (4) and the gut microbiota has an intricate relationship with this chronic low-grade inflammatory response (5). IL-1β is among the pro-inflammatory cytokines of the IL-1 family, and its levels can increase in hypertensive patients (35, 36). Hence, we also explored the impact of IH on serum IL-1β levels in T2DM mice and their association with blood biochemical indices and gut microbiota.

Growing evidence suggests that SCFAs play an important role in the maintenance of gut health and glycolipid metabolism. In the present study, butyric, isobutyric, and valeric acid levels in the cecal contents of the T2DM model group mice were lower than those of the NC group mice, while the IH75 and IH150 groups exhibited significantly higher levels of all three acids. These results suggested that IH can help restore the gut health of patients with T2DM. Correlation analysis between SCFAs and *Lactobacillus* revealed that higher levels of acetic, butyric, and valeric acid were accompanied by lower *Lactobacillus* abundance. A reason for this might be that the T2DM mice experienced a significant increase in *Lactobacillus* abundance, with IH intervention reversing this change. SCFAs are the end products of resistant starch or dietary fiber fermentation by intestinal microbiota and cannot be completely hydrolyzed by the human digestive system due to a lack of specific enzymes (37). SFCAs act as signaling molecules between the gut microbiota and pancreas either directly *via* receptors on pancreatic cells or through the gut-brain-pancreas axis, increasing glucagon-like peptide-1 levels that regulate pancreatic insulin and glucagon secretion and thereby improve glucose homeostasis and insulin sensitivity in patients with T2DM (38). The most abundant SCFAs in the human body are acetate, propionate, and butyrate (39). Butyrate is a well-known histone deacetylase inhibitor that promotes β-cell development, proliferation, differentiation, and function (40). Further, propionate and butyrate suppress inflammation by downregulating pro-inflammatory cytokines (i.e., TNF-α, IL-1β, and IL-6) (41) and can effectively inhibit NF-κB pathway activation as well as cytokine release *in vitro* (42). Hence, we explored the impact of IH on SCFA levels.

Further, IH can improve insulin resistance associated with T2DM (17). In a previous study (43), IH lowered the fasting blood glucose levels, renal condition, and blood lipid profile of T2DM rats by upregulating autophagy in renal tissues. A previous study suggested that cactus extracts rich in IH glycosides can prevent the development of dietary-induced obesity-related metabolic abnormalities (16). Further, IH at a concentration of 20 mg/kg reduced reactive oxygen species levels and macrophage apoptosis, inhibiting atherosclerotic plaque formation in mice (44). In another study, 100 mg/kg of IH inhibited the PI3K/AKT signaling pathway and reduced myocardial hypertrophy as well as fibrosis caused by pressure load (45). After considering the above findings, IH was selected as the intervention agent in our study, and three dosage groups, (low, medium, and high), were used to determine its therapeutic effects in a mouse model of T2DM. The results showed that the medium dose group had a better anti-diabetic effect on T2DM mice.

Animal studies on fecal microbiota transplantation suggested that the relative abundance of Firmicutes was greater in obese leptin-deficient mice compared to lean control mice (46). In the current study, intervention with low or medium doses of IH lowered the proportions of Firmicutes and Bacteroidetes, suggesting that IH may improve lipid metabolism in patients with T2DM. Correlation analyses revealed that increased *Bacteroidales S24-7 group_norank* abundance was associated with lower levels of the inflammatory cytokine IL-1β and cholesterol, suggesting that *Bacteroidales S24-7 group_norank* may protect mice against T2DM. In our experiment, all groups, excluding the NC group, exhibited significantly greater *Lactobacillus* abundance than the NC group, but the mechanism underlying this increase of *Lactobacillus* in T2DM remains unclear. Thus, further studies need to be conducted to elucidate the link between *Lactobacillus* abundance and lower levels of acetic, propionic, and butyric acids.

Principal component analysis is a technique for simplified data analysis that can be used to effectively identify the most dominant elements and structures in data, eliminate noise and redundancy, reduce the dimensionality of the original complex data, and reveal the simple structures hidden behind complex data. For example, the more similar the sample composition, the closer the distance reflected in the PCA plot (47). In our study, IH increased the abundance and diversity of gut microbiota in the T2DM model mice, improving community structure.

In conclusion, IH can improve dyslipidemia in T2DM mice, reduce the expression of inflammatory cytokines, and promote the health of type 2 diabetic mice. This improvement may be related to the decrease of *Lactobacillus* and the increase of *Bacteroidales S24-7 group_norank* and the SCFAs. However, mouse experiments are not enough, we still need more basic research to verify its pharmacological and toxicological effects before gradually move into clinical trials.

## Data availability statement

The data presented in this study are deposited in the NCBI repository, accession number: PRJNA827863.

## Ethics statement

This animal study was reviewed and approved by the Zhejiang Academy of Agricultural Sciences.

## Author contributions

JL, XW, PS, LZ, and YX: conceptualization. XL and LZ: methodology. HY, XL, LL, and LZ: investigation. JL, HY, and LZ: data curation. JL, HY, LL, and XW: formal analysis. JL, ZZ, YX, and HY: writing – original draft preparation. PS, YX, LL, and LZ: writing – review and editing. JL and LL: funding acquisition. All authors contributed to the article and approved the submitted version.
