# Supplementary material for: The mitigative effect of isorhamnetin against type 2 diabetes via gut microbiota regulation in mice
Source: Front Nutr. 2022 Dec 22;9:1070908. doi: 10.3389/fnut.2022.1070908 (PMC9815710; doi:10.3389/fnut.2022.1070908)
Supplement: Supplementary file 1 [file Data_Sheet_1.PDF]

Supplementary Material

Supplementary Figures

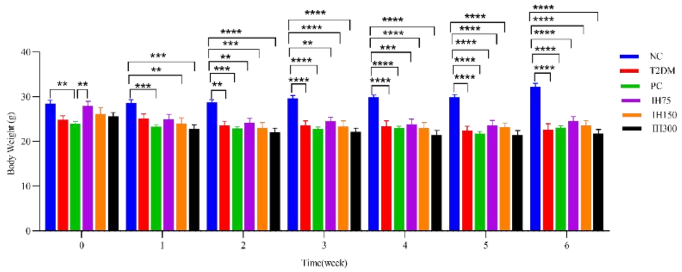

Figure S1. Effects of IH on body weight. All data are expressed as mean ± standard error of the mean (SEM). n=6, P<0.05(*), P<0.01 (**), P<0.001 (***), and P<0.0001 (****).

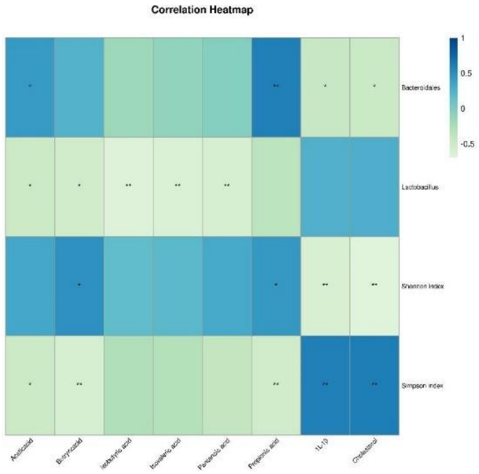

Figure S2. Correlation analysis of short-chain fatty acids, biochemical indicators and intestinal flora. All data are expressed as mean ± standard error of the mean (SEM). n=6, P<0.05(*), P<0.01 (**).

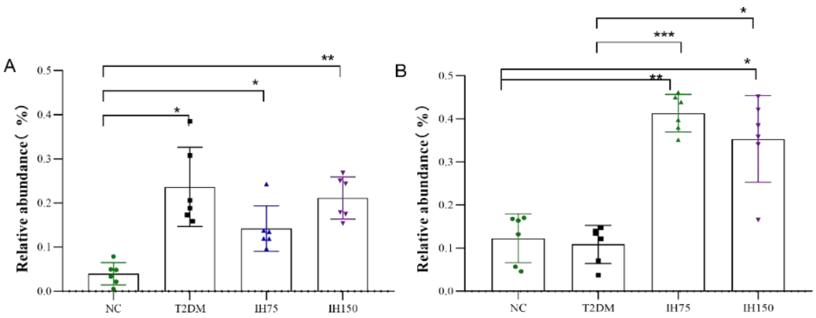

Figure S3. Analysis of the intestinal flora. All data are expressed as mean ± standard error of the mean (SEM). n=6, P<0.05(*), P<0.01 (**), P<0.001 (***), and P<0.0001 (****).T2DM: type 2 diabetes mellitus.
